# Isolation of Bioactive Compounds, Antibacterial Activity, and Action Mechanism of Spore Powder From *Aspergillus niger* xj

**DOI:** 10.3389/fmicb.2022.934857

**Published:** 2022-07-11

**Authors:** Longfeng Wei, Qinyu Zhang, Ailin Xie, Yang Xiao, Kun Guo, Shuzhen Mu, Yudan Xie, Zhu Li, Tengxia He

**Affiliations:** ^1^Key Laboratory of Plant Resource Conservation and Germplasm Innovation in Mountainous Region (Ministry of Education), College of Life Sciences, Institute of Agro-Bioengineering, Guizhou University, Guiyang, China; ^2^Guizhou Key Laboratory of Agricultural Biotechnology, Guiyang, China; ^3^Institution of Supervision and Inspection Product Quality of Guizhou Province, Guiyang, China; ^4^State Key Laboratory of Functions and Applications of Medicinal Plants, Guizhou Medical University, Guiyang, China

**Keywords:** *Aspergillus niger* xj, isolation and identification, bioactive compounds, antibacterial activity, minimum inhibitory concentration, antibacterial mechanism

## Abstract

*Aspergillus* fungi can produce a wide range of secondary metabolites, and they have represented a potential resource of novel bioactive compounds. Bacterial plant diseases have a serious impact on the sustainable development of agriculture worldwide, so it is necessary to use natural antibacterial compounds in microorganisms to control plant pathogens. This study was conducted to investigate the bioactive compounds of *Aspergillus niger* xj, three plant pathogens (*Agrobacterium tumefaciens* T-37, *Erwinia carotovora* EC-1, and *Ralstonia solanacearum* RS-2) were used as indicator bacteria, according to the biological activity tracking, five compounds were isolated from *A. niger* xj spore powder, and characterization of compounds was done by NMR (^1^H-NMR and ^13^C-NMR) and EI-MS and was identified as ergosterol (1), β-sitosterol (2), 5-pentadecylresorcinol (3), 5-hydroxymethyl-2-furancarboxylic acid (4), and succinimide (5). Compounds 3 and 5 were isolated from *A. niger* xj for the first time. The minimum inhibitory concentration (MIC) of five compounds against three plant pathogens was evaluated, the results showed that compound 4 exhibited the strongest antibacterial activity against tested bacteria, and RS-2 was the most sensitive to compound 4, showing the lowest MIC of 15.56 μg/ml. We concluded that the mechanism of action of the compound 4 against RS-2 might be described as compound 4 acting on bacterial protein synthesis and intracellular metabolism according to the results of the scanning electron microscopy observation, permeability of cell membrane and SDS-PAGE. These results indicated that compound 4 has good potential to be as a biocontrol agent. In conclusion, the results from this study demonstrated that the compounds with antibacterial activity are of great significance of the prevention and control of plant phytopathogenic bacteria, and they may be applicable to exploring alternative approaches to integrated control of phytopathogens.

## Introduction

Bacterial pathogens can cause multiple plant diseases, and plant pathogens of food crops are a major constraint on agricultural production worldwide, which can cause significant damage to crop species and consequently food shortages and economic loss (Henry et al., 2013). *Erwinia carotovora* is a member of the family *Enterobacteriaceae*, and it is also a widespread gram-negative plant pathogen, which could affect crops and other plants in subtropical and temperate regions and has a wide host range that includes brussels sprout, carrot, celery, cucumber, capsicum, turnip, and potato (Avrova et al., 2002; Toth et al., 2003). This soil-borne facultative anaerobic bacterium causes maceration and rotting of parenchymatous tissue of all plant organs, resulting in the loss of the entire plant (Snijder and Tuyl, 2002). *Agrobacterium tumefaciens* is a gram-negative bacterium of the genus *Agrobacterium* (Fuller et al., 2017). *A. tumefaciens* can infect 643 species of dicotyledonous plants and a few gymnosperm plants of 331 genera and 93 families (Gao, 2015). *Ralstonia solanacearum* is a species complex due to the differences in geographic origin, host range, and pathogenic behavior (Denny, 2007), and the current taxonomic classification of *R. solanacearum* species complex (RSSC) includes three different species, including *R. solanacearum* (phylotype II), *R. pseudosolanacearum* (phylotypes I and III) and *R. syzygii* (phylotype IV) (Fegan and Prior, 2005; Castillo et al., 2020). *Ralstonia solanacearum* is a destructive soil-borne plant pathogen, which is widely distributed in tropical, subtropical, and temperate regions (Peng et al., 2021). It has a very broad host range that can infect more than 450 species in 54 botanical families (Wicker et al., 2007), and it is the causal agent of potato brown rot, bacterial wilt of tomato, tobacco, eggplant, and some ornamentals, as well as Moko disease of banana (Mansfield et al., 2012). Due to its wide geographical distribution and host range, the impact of *R. solanacearum* on agricultural production is difficult to quantify; on potato alone, it has been reported to cause losses of approximately 1 billion dollars each year worldwide (Elphinstone, 2005). *R. solanacearum* infects host plants through plant root wounds or root tip cracks, invades the xylem vessels and spreads rapidly to aerial parts of the plant through the vascular system (Genin, 2010), grows to high cell density and produces a large number of extracellular polysaccharides, hinders water transport, and leads to plant wilting and final death (Yang et al., 2021). In conclusion, the three common plant pathogens have caused greater economic losses than other bacterial diseases in the world, and *A. tumefaciens* and *R. solanacearum* were placed in a top 10 bacterial plant pathogen list in 2012 (Mansfield et al., 2012), which is considered to pose a serious threat to agricultural production. It has been reported that bacterial pathogens can directly or indirectly cause an estimated 40 billion dollars in losses worldwide every year (Syed Ab Rahman et al., 2018).

At present, antibiotics and chemicals are mainly used to control plant diseases in agricultural production, but the long-term use of chemical pesticides will produce a series of side effects, including improving the drug resistance of plant pathogens, causing crop pesticide residues, environmental pollution, endangering human health, and destroying the ecological balance (Pandit et al., 2022). Breeding resistant varieties is the most reliable management tool for controlling bacterial diseases, despite the characteristic potential to provide disease resistance to the host, the use of genetically modified crops remains the subject of global controversy (Scott et al., 2016). With the development of biotechnology, people began to look for a plant disease control strategy that is harmless to humans and the environment. Therefore, biological control has attracted more and more attention because of its good biological control effect, non-toxic, harmless, and pollution-free characteristics (Koch et al., 2018), and beneficial biocontrol microbes may be one of the few options with potential (Syed Ab Rahman et al., 2018).

Fungi can produce a wide range of secondary metabolites; therefore, they have represented a potential resource for the discovery of novel bioactive molecules (Boustie and Grube, 2005). Members of the genus *Aspergillus* are well known to produce chemically diverse secondary metabolites (Ding et al., 2019), including pyranone, alkaloid, cyclopentapeptide, polyketide, sterol, etc., which have antibacterial, anticancer, antioxidant, antiviral, and other functions (Swathi et al., 2013; Holm et al., 2014; Leutou et al., 2016; Akinfala et al., 2020; Jomori et al., 2020; Padhi et al., 2020). Liu et al. (2017) isolated diphenyl ether from *Aspergillus sydowii*, and it had antibacterial activity against *Staphylococcus aureus*, *Streptococcus iniae*, and *Vibrio ichthyoenteri*. An et al. (2019) isolated the compound emodin from a marine fungus *Aspergillus* sp. SCS-KFD66. The activity test results showed that the MIC of emodin against *S. aureus* and *Bacillus subtilis* was 16 and 64 μg/ml, respectively. Therefore, *Aspergillus* fungi are one of the important sources for the discovery of active lead compounds and novel structures, which is of great significance for the study of antibacterial secondary metabolites of *Aspergillus*.

*Aspergillus niger* is a filamentous ascomycete fungus that widely exists in the environment (Baker, 2006). In the natural growth state, *A. niger* obtains nutrients by secreting numerous multi-purpose enzymes to degrade biopolymers in the environment. In addition, *A. niger* has been recognized as a safe strain by the Food and Drug Administration (FDA) of the United States because of its strong metabolism, fast growth, and short fermentation cycle (Pel et al., 2007). It is widely used to produce organic acids, proteins and enzymes, and other substances (Abarca et al., 2004; Steiger et al., 2019). Therefore, *A. niger* strains are used as cell factories for enzyme production by many biotechnology firms (Meyer et al., 2016). In the early stage of the laboratory, Li et al. (2011) isolated a strain of *A. niger* xj from plant rhizosphere soil, and its fermentation broth had varied degrees of inhibitory effect on five common pathogenic fungi. In addition, the previous laboratory results showed that *A. niger* xj has good inhibitory effects on bacteria. Guo et al. (2021) showed that the crude extract (B10) of *A. niger* xj spores had effective antibacterial activity against *A. tumefaciens* T-37, with an inhibition rate of 98.22%; Liu et al. (2016) found that the crude extract of *A. niger* xj spore powder showed good inhibitory effects on *R. solanacearum*, and the inhibitory rate was greater than 92.02%. Therefore, in our previous studies, *A. niger* xj is considered to be a potential source of antibacterial bioactive compounds.

The objective of this study was to isolate and characterize antibacterial bioactive compounds from *A. niger* xj spore powder against plant pathogens, including *Agrobacterium tumefaciens* T-37, *Erwinia carotovora* EC-1, and *Ralstonia solanacearum* RS-2, and the antibacterial activities of monomeric compounds against three indicator bacteria were evaluated by measuring the MIC values. The potential antibacterial mechanisms responsible for the antibacterial activity against sensitive strains were determined by scanning electron microscopy (SEM) observation, permeability of cell membrane, and sodium dodecyl sulfate–polyacrylamide gel electrophoresis (SDS-PAGE), which may help to develop valuable microbial-derived pesticides in agriculture.

## Materials and Methods

### Strains

The *Aspergillus niger* xj was isolated and identified by the Institute of Fungi Resource, Guizhou University and stored at the China Center for Type Culture Collection (CCTCC), CCTCC no. M206021, patent number ZL 2006 10051022.6.

*Ralstonia solanacearum* RS-2 belongs to phylotype II, sequevar 1 (race 3, biovar 2), and it was preserved by the Microbiology Laboratory of Guizhou University.

*Agrobacterium tumefaciens* T-37 was purchased from the Soil and Fertilizer Institute of the Chinese Academy of Agricultural Sciences and stored in the Institute of Fungi Resource, Guizhou University.

*Erwinia carotovora* EC-1 was isolated, screened, identified, and preserved by the Laboratory of Fungal Resources Institute of Guizhou University.

### Culture Media

Beef extract peptone liquid medium: 10 g/L peptone, 5 g/L NaCl, 3 g/L beef extract, and 1 L of distilled water at pH 7.4–7.6. The medium was used to culture three indicator plant pathogens to guide the fractionation of active fractions and investigate the antimicrobial activity of the compounds.

Beef extract peptone solid medium: 15–20 g/L of agar was added to beef extract peptone liquid medium. The medium was used to culture three indicator bacteria to obtain active strains.

Potato dextrose agar (PDA) medium: 200 g/L of potato, 20 g/L of glucose, 15–20 g/L of agar, and 1 L of distilled water at natural PH. The medium was used for the culture of *A. niger* xj strain.

Potato dextrose broth (PDB) medium: PDA medium without agar, which was used to prepare seed broth of *A. niger* xj strain.

Solid-state fermentation medium: 1 g/L NaNO_3_, 131.2 g/L sucrose, wheat bran: distilled water (8:8, m/v), the initial pH was adjusted to 7.2. The medium was used for solid fermentation of *A. niger* xj strain to obtain higher sporulation yields. All media were autoclaved at 121°C for 20 min.

### Fermentation

The *A. niger* xj strain was cultured on a PDA plate at 28°C for 7 days. The spore of the fungus was collected and mixed with PDB medium to make a spore suspension (1 × 10^5^ spores/ml), and 10% (v/m) of *A. niger* xj spore suspension was inoculated into the solid-state fermentation medium, performed shallow plate fermentation, and cultivated in light at 28°C for 7 days. After spore production, the solid matrix in each material plate was transferred to an oven at 33°C for drying for 24–48 h, and the dried matrix was ground into particles, then separated, and collected the *A. niger* xj spore powder, and the spore production measured by hemocytometer was 2 × 10^10^ spores/g.

RS-2, EC-1, and T-37 were cultured on the solid medium of beef extract peptone at 30°C for their respective logarithmic phase (RS-2 16 h, EC-1 24 h, and T-37 18 h) to activate the strains (Liu et al., 2016). The activated strains were incubated in 25 ml of beef extract peptone liquid medium with continuous shaking (150 rpm/min) at 30°C to their logarithmic phase, the bacterial culture was centrifuged at 6,000 rpm for 10 min to prepare bacterial suspension with sterile water, and the concentration was adjusted to 10^8^ CFU/ml.

### Extraction and Isolation of Spore Powder of *A. niger* xj

**FIGURE 1:**
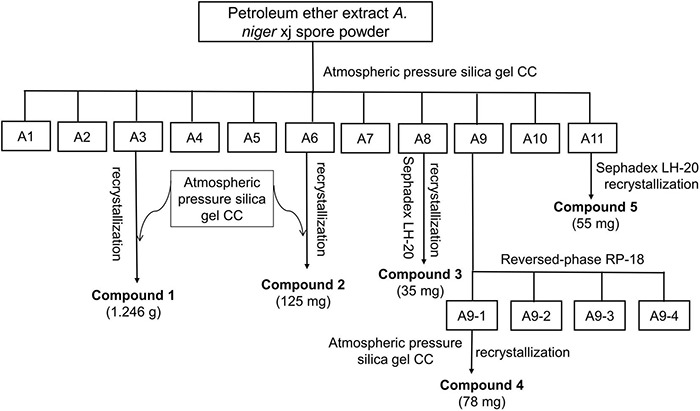
The antibacterial activity guided fractionation of petroleum ether extract.

The *A. niger* xj spores obtained after fermentation were made into pulverized powder. About 5 kg of the powder was reflux extracted three times with ethanol (each 15 L) at 80°C for 4 h, and then, the total ethanol extract was filtered with 8 layers of gauze and concentrated at 40°C with a rotary evaporator to obtain the crude ethanol extract. To obtain petroleum ether, ethyl acetate, and water fractions, 100 g of crude ethanol extract was dissolved in 200 ml of distilled water and then sequentially extracted three times each with 200 ml of petroleum ether, ethyl acetate, and water. The combined organic extracts were concentrated and dried under reduced pressure. The crude fractions of petroleum ether were separated under the guidance of antibacterial activity: the petroleum ether extract (445 g) was separated by silica gel column chromatography (CC) and subjected with different solvent systems to gradient elution such as pure petroleum ether, petroleum ether-ethyl acetate solution (petroleum ether-ethyl acetate = 100:1, 50:1, 20:1, 10:1, 5:1, 2:1, 1:1, v/v), chloroform-acetone solution (chloroform: acetone = 20:1, 10:1, 5:1, 2:1, 1:1, v/v), and pure methanol. Fractions (200 ml) were collected, concentrated, and merged with the fractions having the same polarity composition as determined by thin layer chromatography (TLC) analysis. The TLC results of the crude extract indicated that the extraction contained a variety of active compounds with different bioactivities. Therefore, further separation was needed to get more compounds. Finally, 11 fractions (A1–A11) were obtained from this separation process (Figure 1). The antimicrobial activity-guided fractionation of bioactive fractions A3, A6, A8, A9, and A11 was performed as follows:

A3 fraction was first separated by atmospheric pressure silica gel CC (200–300 mesh), eluting with petroleum ether-ethyl acetate (20:1, 10:1, 5:1, v/v), collected the fraction (10:1, v/v), and further purified by atmospheric pressure silica gel, using petroleum ether-ethyl acetate (6:1, v/v) to afford compound 1 (1.246 g). Recrystallization in petroleum ether-methanol (50:1, v/v) was performed to obtain a pure compound. A6 fraction was separated by atmospheric pressure silica gel CC (200–300 mesh) with petroleum ether-ethyl acetate (10:1, 5:1, 2:1, v/v) as eluent. Subfraction (5:1, v/v) was acquired and then using petroleum ether-ethyl acetate (50:1, v/v) to recrystallize to give compound 2 (125 mg). A8 fraction was chromatographed over silica gel, using petroleum ether-ethyl acetate (2:1, 1:1, v/v) as a mobile phase, subfractions (1:1, v/v) were collected and further separated by Sephadex LH-20, eluting with a gradient of chloroform-methanol (1:1, v/v), and fractions were collected which produced spot at *Rf* 0.3 when eluted by petroleum ether-ethyl acetate (8:1, v/v) to obtain compound 3 (35 mg). Recrystallization in petroleum ether-ethyl acetate (50:1, v/v) was performed to obtain pure compound at 4°C. A9 fraction was subjected to fractionation over reversed-phase silica gel CC, eluting with elution gradient of H_2_O-acetone (1:0, 9:1, 8:2, 7:3, 6:4, 5:5, 4:6, 3:7, 2:8, 1:9, 0:1) to give four subfractions A9-(1–4). Subfraction A9-1 (3.582 g) was further subjected to column chromatography using an atmospheric pressure silica gel (200–300 mesh) and eluted using a gradient of petroleum ether-acetone (5:2, v/v), and the subfraction was further purified by silica gel CC with petroleum ether-acetone (5:2, v/v) as eluent to get compound 4 (78 mg). A11 fraction was subjected to Sephadex LH-20 and eluted with chloroform-methanol (1:1, v/v). Fractions, which produced a spot at *Rf* 0.3 when eluted with petroleum ether-acetone (2:1, v/v), were collected and further separated Sephadex LH-20 to result in compound 5 (55 mg), which was recrystallized at 4°C with petroleum ether-methanol (50:1, v/v) to provide the pure compound.

### Antibacterial Activity

T-37, EC-1, and RS-2 strains were used as the indicator bacteria, and the experiment evaluated the antibacterial activity of the organic extracts and active fractions against them. Generally, these organic extracts and active fractions are separately dissolved in dimethyl sulfoxide (DMSO) to a concentration of 100 mg/ml. Then, 10 μl of the solutions was added to 1 ml of beef extract peptone liquid medium to obtain a final concentration of 1 mg/ml, and 100 μl of each bacterial suspension (10^8^ CFU/ml) was inoculated and incubated at 30°C and 150 rpm for their logarithmic phases. Chloramphenicol (CHL) and DMSO were used as the positive control and the solvent control, respectively. All tests were performed in triplicate. Cell density was monitored OD_λ *max*_ using a UV-Vis Spectrophotometer BioMate™ 3S (Thermo, United States) (The OD_λ *max*_ of T-37, EC-1 and RS-2 are 400, 490, and 420 nm, respectively). The calculation formula for the inhibition rate is shown below:


$$I_{R}=\frac{OD_{0}-OD_{1}}{OD_{0}}\times 100\%$$

where *I*_*R*_ is the bacterial inhibition rate, OD_1_ is the OD_λ *max*_ of the experimental group, and OD_0_ is the OD_λ *max*_ of the blank control group separately.

### Identification Compounds

The chemical structure of the monomer compound was identified by liquid chromatograph mass spectrometer HP6890/5975C (Agilent, United States), electron ionization mass spectrometry HP 5973 (Agilent, United States), and nuclear magnetic resonance (NMR) spectroscopy DRX-500 (Bruker, Switzerland). Mass spectrometry analysis of the compounds was performed following the method of Diao et al. (2014a). NMR was performed by referring to the analytical method of Arora and Itankar (2018). The obtained data were compared with the data of the known compounds in the literature to determine the structure of the compounds.

### Determination of the Minimum Inhibitory Concentration

Determination of antimicrobial properties of (bio)molecules has been conventionally performed using minimum inhibitory concentration (MIC) (Dudu et al., 2021). The MIC values were defined as the lowest concentration at which there is no visible turbidity (Mawire et al., 2021), and the MIC was determined by broth microdilution assay as described by Xie et al. (2021) with some modifications. T-37, EC-1, and RS-2 strains were incubated at 30°C for logarithmic phase to approximately 10^8^ CFU/ml in beef extract peptone liquid medium previously. The compounds were dissolved in DMSO (1 mg/ml) and diluted at concentrations of (500, 250, 125, 62.5, 31.25, 15.63, 7.82, 3.91, 1.96, 0.98, 0.49, and 0.25 μg/ml) by 2-fold dilutions on a 96-well plate. About 100 μl of each bacterial suspension (10^8^ CFU/ml) was inoculated in each well. Microplates were incubated at 30°C and the MICs were recorded after 24 h of incubation. DMSO and CHL were used as the solvent control and the positive control, respectively. All tests were performed in triplicate.

### Antibacterial Mechanism of Compound 4 Against RS-2

#### Determination of Half Maximal Effective Concentration (EC50)

Serial 2-fold dilutions of compound 4 were prepared in beef extract peptone liquid medium at concentrations of 1.0000, 0.5000, 0.2500, 0.1250, 0.0625, and 0.0313 mg/ml. RS-2 suspension was added into all the tubes to achieve an initial inoculum of approximately 1 × 10^8^ CFU/ml. All tubes were incubated at 30°C, 150 rpm for 24 h, and the OD value was measured to calculate the inhibition rate. Methanol and CHL were used as the solvent control and the positive control, respectively. All experiments were performed in triplicate.

#### Scanning Electron Microscopy Analysis

Scanning electron microscopy observation on the tested bacteria was performed according to the method described by Zhang et al. (2020) with some modifications. About 200 μl of cell suspension was added to 20 ml of fresh culture medium, and then, the suspension was added EC50 concentration of the compound 4. All suspensions were incubated at 30°C, 150 rpm for 6 h and then centrifuged at 5,000 rpm for 5 min. The cells were washed three times with 0.1 M PBS (pH 7.4) and fixed with 2.5% (v/v) glutaraldehyde in 0.1 M PBS overnight at 4°C, and then, the cells were centrifuged at 8,000 rpm for 3 min, repeated three times. After this, the cells were further dehydrated using a graded series of ethanol (30, 50, 70, 80, 90, and 100%), and then, the ethanol was replaced by tertiary butyl alcohol for 10 min. Finally, cells were dried and gold-covered by cathodic spraying, and morphology of the bacterial cell was observed with a scanning electron microscopy (Hitachi S-3400N, Hitachi, Japan).

#### Permeability of Cell Membrane

The permeability of the cell membrane is expressed as the relative electrical conductivity, which was determined according to the method described by Diao et al. (2014b). Cells of bacteria were prepared and treated as described in Section “Fermentation.” About 200 μl of cell suspension was added to 20 ml of fresh culture medium, and compound 4 at EC50 concentration was added to bacteria solution. After completely mixed, the samples were incubated at 30°C and shaken with a rotary shaker at 150 rpm. During the culture, six samplings were carried out at 1, 2, 3, 4, 5, and 6 h, and all samples were centrifuged at 8,000 rpm for 3 min and measured the conductivity in the supernatant. CHL was used as the positive control, and the experiments were performed in triplicate.

#### Sodium Dodecyl Sulfate–Polyacrylamide Gel Electrophoresis of Whole-Cell Proteins

Sodium dodecyl sulfate–polyacrylamide gel electrophoresis of the bacterial proteins was carried out according to the method described by Zhao et al. (2015) with some modifications. About 300 μl of cell suspension was added to 30 ml of fresh culture medium in the presence of compound 4 at the EC50 concentration, and the blank control was prepared as described above, but in the absence of compound 4, CHL was used as the positive control. After completely mixed, all samples were incubated at 30°C, 150 rpm for 12 h. During the culture, four samplings were carried out at 0, 4, 8, and 12 h, and 1 ml of each sample was collected and centrifuged at 8,000 rpm for 5 min. Bacterial cells were washed three times with 0.1 M phosphate buffer saline (PBS, pH 7.2) and resuspended in PBS at the same original volume. After that, all cell suspensions (the treated and control groups) were adjusted to the same cell density (OD_420_). An aliquot of 25 μl of the bacterial suspension was combined with 25 μl of the sample buffer (pH 6.8; 1 M Tris–HCl, 50% glycerol, 10% SDS, 10% β-mercaptoethanol, and 0.1% bromophenol blue) and heated at 100°C for 10 min, and SDS-PAGE was performed. The SDS-PAGE was performed with a 12% separating gel and a 5% stacking gel followed by Coomassie brilliant blue staining for 3 h, and overnight decolorization and protein electrophoresis were recorded by the gel imaging system.

### Statistical Analysis

All experiments were performed in triplicate and expressed as mean ± standard deviation (SD). One-way analysis of variance (ANOVA) was performed for data analysis using IBM SPSS version 22.0. Conversion of SDS-PAGE grayscale values was performed using Image J 17.0 software. The differences among groups were evaluated by performing Duncan’s test, and *p* < 0.05 was considered significant.

## Results

### Antibacterial Activity of Crude Extract and Active Fractions of *A. niger* xj Spore Powder

**FIGURE 2:**
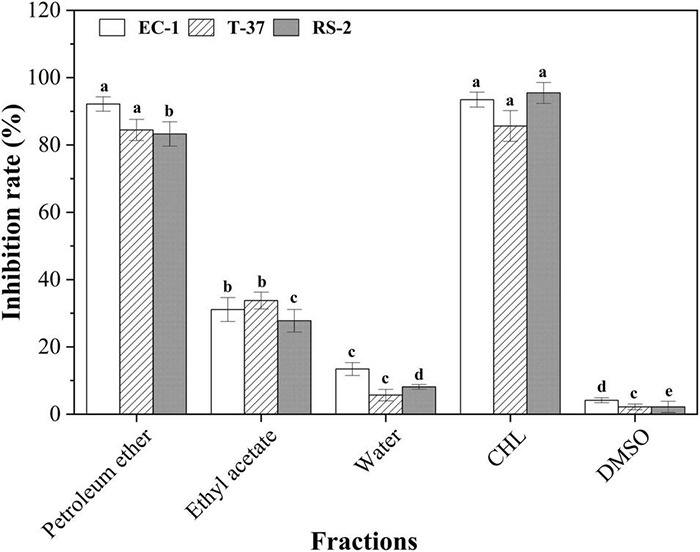
Antibacterial activities of the organic extract fractions of the spore powder against the indicator bacteria. Different lowercase letters indicate significant differences (*p* < 0.05). T-37, EC-1, and RS-2 represent *Agrobacterium tumefaciens* T -37, *Erwinia carotovora* EC-1, and *Ralstonia solanacearum* RS-2, respectively. DMSO represents dimethyl sulfoxide. CHL represents chloramphenicol.

As shown in Figure 2, the results of antibacterial activity of the organic fractions from spore powder of *A. niger* xj indicated that all of the fractions had activity to some extent to the indicator bacteria growth, among them, fraction petroleum ether showed the strongest bioactivity when compared to the solvent control group, and ethyl acetate fractions also showed promising activity against the indicator bacteria (*p* < 0.05). To be specific, the inhibition rates of petroleum ether fraction against RS-2, EC-1, and T-37 were 83.26, 92.15, and 84.45%, respectively, and our data suggested that the antibacterial activity of petroleum ether fraction against EC-1 and T-37 strains was comparable to the positive control group (*p* > 0.05). In addition, the inhibition rates of ethyl acetate fraction on three indicator bacteria were less than 40%, and the water phase showed the weakest antibacterial activity against the indicator. Based on the comprehensive analysis of the polarity of the three-stage eluent, it was preliminarily speculated that the antibacterial bioactive compounds in the crude extract of *A. niger* xj spore powder mainly present in low polarity fraction. In conclusion, the results from this study demonstrated that petroleum ether was the optimum extraction solvent. Therefore, the petroleum ether fraction was selected as the separation center of gravity of the bioactive compound.

**FIGURE 3:**
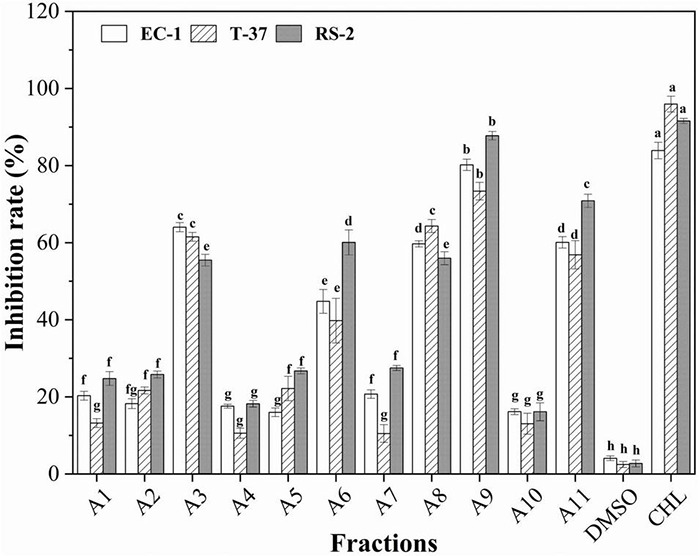
Antibacterial activities of the subfractions A1–A11 of the spore powder against the indicator bacteria. Different lowercase letters indicate significant differences (*p* < 0.05).

A total of eleven fractions obtained from the petroleum ether fraction were screened to determine the distribution of bioactive compounds (Figure 3). The results clearly showed that five subfractions A3, A6, A8, A9, and A11 exhibited strong antibacterial activities against the indicator bacteria. In particular, A6, A9, and A11 fractions showed stronger antibacterial activities than other fractions against RS-2, their inhibition rates were 60.07, 87.75, and 70.83%, respectively, and it is highly probable that bioactive compounds were present in the three organic fractions. A3, A8, A9, and A11 showed strong antibacterial activity against EC-1 and T-37, indicating that a large number of antibacterial bioactive compounds against them present in these fractions, especially in A9. Furthermore, it can be observed that A6 had different degrees of antibacterial activity against EC-1 and T-37, among which the inhibition rates were less than 50%. Therefore, antibacterial activity-guided fractionation of A3, A6, A8, A9, and A11 subfractions was performed for further separation and purification. Finally, compounds 1–5 were obtained from the A3, A6, A8, A9, and A11 fractions, respectively.

### Identification Compounds

**FIGURE 4:**
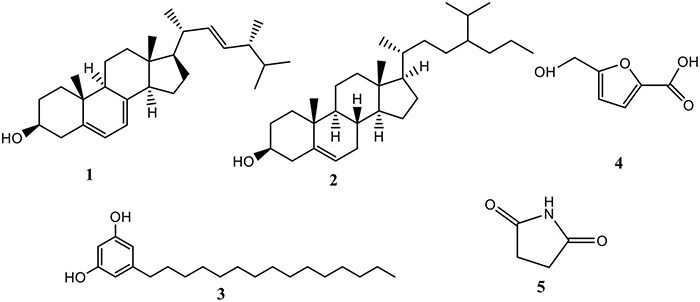
The chemical structures of compounds isolated from *A. niger* xj.

The chemical structure of all compounds was characterized by EI-mass spectrometry and NMR (^1^H and ^13^C) as described below. A total of five compounds were isolated in the fractions of extracts from the spore powder of *A. niger* xj, which were identified as ergosterol (1), β-sitosterol (2), 5-pentadecylresorcinol (3), 5-hydroxymethyl-2-furancarboxylic acid (4), and succinimide (5) (Figure 4).

Compound 1: C_28_H_44_O, white needle-shaped crystal. EI-MS (m/z): 396[M]^+^; ^13^C-NMR (400 MHz, Pyridine-D5) δ: 38.89, 33.02, 69.91, 42.00, 140.89, 117.16, 119.66, 141.32, 46.62, 37.50, 21.43, 39.35, 43.12, 54.83, 23.41, 28.78, 55.85, 12.24, 16.59, 43.05, 21.40, 136.15, 132.16, 40.85, 33.37, 19.89, 20.20, 17.89. ^1^H-NMR (400 MHz, Pyridine-D5) δ: 5.72, 5.51, 5.28, 3.97, 2.83, 2.68, 2.16, 2.04, 1.90, 1.75, 1.59, 1.55, 1.47, 1.37, 1.25, 1.11, 1.10, 1.06, 1.00, 0.98, 0.89, 0.86, 0.87, 0.69 (Supplementary Figures 1–3). Compound 1 was identified as ergosterol by comparison of its spectral data with those reported in the study of Shirane et al. (1990).

Compound 2: C_30_H_52_O, white powder. EI-MS (m/z): 414[M]^+^; ^13^C-NMR (151 MHz, CDCl_3_) *δ:* 140.9, 121.3, 71.1, 56.4, 55.9, 50.1, 46.0, 42.3, 42.2, 39.4, 37.1, 36.5, 36.5, 33.8, 32.1, 31.9, 31.9, 29.1, 26.4, 25.9, 24.4, 23.2, 21.1, 19.4, 19.3, 12.1, 11.9. ^1^H-NMR (600 MHz, CDCl_3_) δ: 5.36, 3.54, 1.03, 0.92, 0.87, 0.83, 0.81, 0.68 (Supplementary Figures 4, 5). Comparing the data in the scientific literature, the structure of the isolated compound 2 was determined to be β-sitosterol (Mao, 2002).

Compound 3: C_21_H_36_O_2_, white solid. EI-MS (m/z): 320[M]^+^. ^13^C-NMR (151 MHz, CDCl_3_) δ: 157.8, 144.9, 106.5, 99.5, 48.1, 47.9, 47.8, 47.6, 47.5, 47.3, 47.2, 35.6, 31.7, 31.1, 29.4, 29.3, 29.1, 29.0, 22.4, 13.1. ^1^H-NMR (600 MHz, CDCl_3_) δ: 6.17, 1.49, 1.18, 0.81. The *Rf* value was consistent with the standard product; based on the spectral data analysis, the structure of compound 3 was identified as 5-pentadecylresorcinol (Zhao et al., 2018; Supplementary Figures 6, 7).

Compound 4: C_6_H_6_O_4_, white crystal. EI-MS (m/z): 396[M]^+^. ^13^C-NMR (125 MHz, CD_3_OD) δ: 162.0, 160.8, 145.9, 120.1, 110.4, 57.6. ^1^H-NMR (600 MHz, CD_3_OD) δ: 7.16, 6.17, 5.07. Comparing with the spectral data in the literature (He et al., 2014), compound 4 was identified as 5-hydroxymethyl-2-furancarboxylic acid (Supplementary Figures 8, 9).

Compound 5: C_4_H_5_NO_2_, white crystal, EI-MS (m/z): 396[M]^+^. ^13^C-NMR (151MHz, CD_3_OD) δ: 207.0, 192.5, 138.8, 136.1, 133.4, 130.9, 129.1, 128.8, 128.7, 128.4, 128.3, 128.2, 117.1, 111.9, 66.5, 66.3, 65.6, 58.3, 30.9, 30.6, 29.7, 19.2, 13.7. ^1^H-NMR (600MHz, CD_3_OD) δ: 10.8, 7.9, 5.2, 4.6, 4.1, 4.1, 3.4, 2.1, 1.3, 1.7. In addition, the *Rf* value was consistent with the standard product. Comparing with the spectral data in the literature (Aqueveque et al., 2002), compound 5 was identified as succinimide (Supplementary Figures 10, 11).

### Determination of the Minimum Inhibitory Concentration

As fungal metabolites have been considered as a promising source of antibiotics, to further compared the antibacterial activities of different compounds against three plant pathogens, the MIC values were compared in Table 1; compared with other compounds, compound 4 exhibited stronger antibacterial activity against EC-1 and RS-2, with MIC values of 31.25 and 15.65 μg/ml, respectively, and it had moderate antimicrobial effect against T-37 (MIC, 250 μg/ml). Both compounds 2 and 3 exhibited moderate antimicrobial effect against EC-1 and RS-2, with MIC values in the range of 125–250 μg/ml, and the two compounds showed weak inhibition on the growth of T-37 (MIC, 500 μg/ml). Furthermore, compounds 1 and 5 displayed weak antibiotic capacity against the three indicator bacteria (MIC, ≥500 μg/ml). In summary, the monomeric compounds tested generally showed only weak to moderate antimicrobial activity, with compound 4 showing strong antibacterial effects. Based on the results of MIC, the antibacterial mechanism of action of compound 4 against RS-2 will be further investigated in this study.

**TABLE 1:** Antibacterial activities of compounds.

Compounds	MIC (μg/ml)		
	EC-1	T-37	RS-2
1	>500	>500	500
2	250	500	250
3	125	500	250
4	31.25	250	15.65
5	500	500	>500
CHL	15.65	3.91	15.65

### Antibacterial Mechanism of Compound 4 Against RS-2

#### Determination of EC50

**FIGURE 5:**
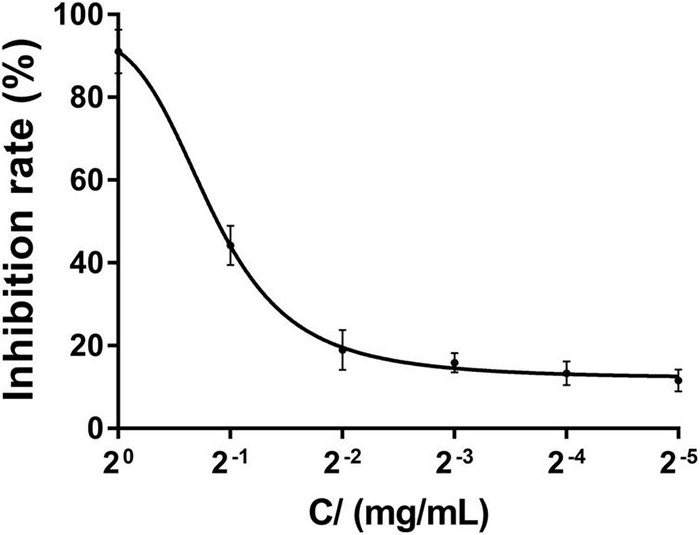
The EC50 value of compound 4 against RS-2.

The results of EC50 determination are presented in Figure 5, and the EC50 value of compound 4 against RS-2 was 0.5611 mg/ml. We can clearly observe that the inhibition rate increased with the increasing concentration of compound 4, and when the concentration gradient reached 1 mg/ml, the inhibition rate had reached more than 80%, and the inhibition effect did not change much when the compound concentration continued to increase.

#### Electron Microscopic Observations

**FIGURE 6:**
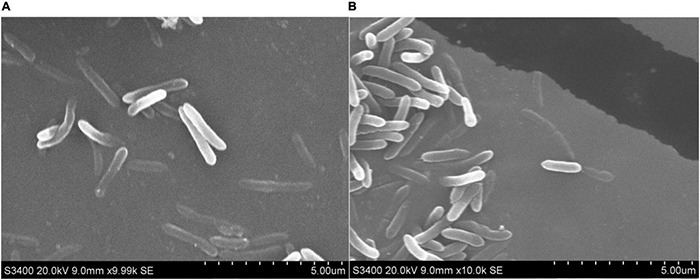
SEM of RS-2 **(A)** untreated cells and **(B)** after treatment with compound 4 at EC50 value for 6 h.

The morphological changes of RS-2 were evaluated by SEM analysis. The electron micrographs of both untreated and compound 4 treated bacterial cells are shown in Figure 6. According to the results, the untreated RS-2 cells had typical cellular organization of gram-negative bacterial with intact cellular structure, the cells treated with compound 4 at EC50 concentration revealed a slightly damaging effect on the cell morphology, showing an obvious apical swelling, and some cells showed irregular morphology compared with the untreated group (Figure 6B). However, compound 4 had less or no effect on the cytolysis of RS-2 cells when viewed by SEM. These findings demonstrated that compound 4 has less effect on the structure and function of RS-2 cell membrane, which may be due to the inhibition of cell activity by affecting cell growth and metabolism or the activity of key enzymes.

#### Cell Membrane Permeability

**FIGURE 7:**
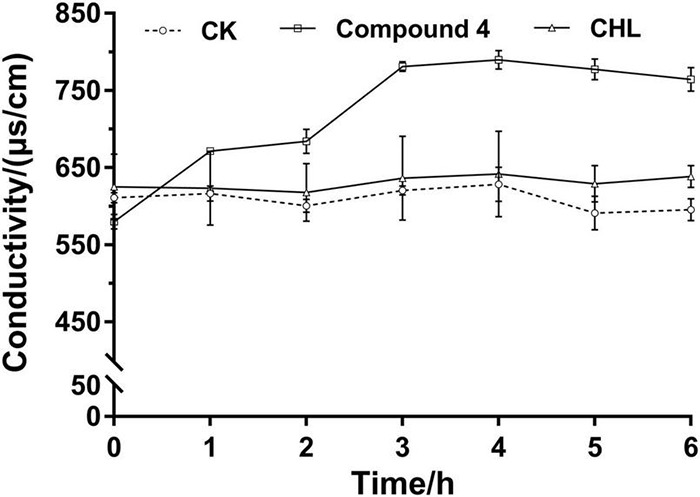
Effect of compound 4 on cell membrane permeability of RS-2.

The results in Figure 7 showed the effect of compound 4 on the membrane permeability of RS-2 by electric conductivity assay. In the control and positive control groups, there was less change in the relative electric conductivity during the whole experiment period. Compared to that of the control, the relative electrical conductivity of the treatment group increased immediately after the addition of EC50 concentration of compound solution, and it also increased rapidly with increasing treatment time within 0–3 h, and the increase of extracellular conductivity tended to be flat at about 3 h. These results indicated that compound 4 has a certain effect on the membrane osmotic function of RS-2, causing leakage of some intracellular ingredients, especially losses of electrolytes, including K^+^, Na^+^, Ca^2+^, and so on (Diao et al., 2014b). The increase of electrolytes in the extracellular environment leads to an increase in extracellular conductivity.

#### Sodium Dodecyl Sulfate–Polyacrylamide Gel Electrophoresis of Whole-Cell Proteins

**FIGURE 8:**
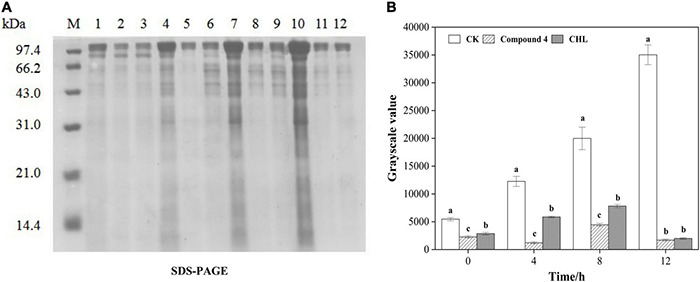
Effect of compound 4 on protein synthesis in RS-2 cells. **(A)** SDS-PAGE of RS-2 cells treated with compound 4. M: Maker; 1, 4, 7, and 10 were the control treatments of 0, 4, 8, and 12 h, respectively. 2, 5, 8, and 11 were treated with compound 4 at 0, 4, 8, and 12 h, respectively. 3, 6, 9, and 12 were treated with chloramphenicol for 0, 4, 8, and 12 h, respectively. **(B)** The grayscale value map of the effect of compound 4 on protein synthesis of RS-2. Different lowercase letters indicate significant differences among treatments at the same exposure time (*p* < 0.05).

The SDS-PAGE profiles of bacterial soluble proteins extracted from RS-2 treated with compound 4 for 12 h are shown in Figure 8A. It can be seen from the figure that in the range of 0–12 h, the protein bands of untreated bacteria showed strong intensities, indicating that the growth of RS-2 was not disturbed. However, the protein profile of bacteria treated with the compound 4 and CHL differed from those of the control from 0 h. Grayscale values indicated that after 0, 4, and 8 h of RS-2 cell culture, there was a significant difference in protein bands between CHL and compound 4 group (*p* < 0.05) (Figure 8B). There were two thick bands (approximately 110 and 90 kDa) in lane 7 for untreated bacteria. However, a band disappeared in lane 8 for treated bacteria with compound 4. In addition, the number of protein bands below 43 kDa in the treated samples was less than blank control and was hardly observed in the lanes. After treatment with compound 4 at EC50 concentration for 12 h, the protein bands faded or even disappeared. Similar results were also found in bacteria cells treated by CHL, and our data indicated that the effect of compound 4 on the protein synthesis of RS-2 strain was comparable to the positive control group (*p* > 0.05). It has been reported that CHL can prevent the extension of peptide chain when messenger RNA translates protein, inhibits protein synthesis, and leads to cell death (Wu et al., 2010). According to Zhao et al. (2015), the reason for the disappearance of protein bands might be that antibacterial compounds interfered with the protein synthesis of bacteria cell or lead to protein leakage from bacterial cells.

## Discussion

Many studies reported that fungi had provided mankind with numerous different bioactive secondary metabolites, and thus, fungi have become an efficient group of organisms to be explored for drug discovery purpose (Swathi et al., 2013). *Aspergillus* spp., including *A. niger*, is rich in enzymes and can produce various types of physiologically active secondary metabolites. As a potential source of novel bioactive compounds, isolation and identification of the chemical constituents of *A. niger* are important. In this study, three plant pathogens such as T-37, EC-1, and RS-2 were used as indicator bacteria to screen the antibacterial compounds extracted from *A. niger* xj spore powder. The petroleum ether fraction of *A. niger* xj spore powder had the best antagonistic activity against RS-2, EC-1, and T-37 strains, and inhibition rates were 83.26, 92.15, and 84.45%, respectively. The polarity of the three eluting solvents is water > ethyl acetate > petroleum ether. It was speculated that antibacterial bioactive compounds in the crude extract of spore powder mainly present in low polarity fractions. Petroleum ether fraction was performed for further separation and purification, obtained eleven fractions, such as A1–A11, and successfully separated five monomeric compounds from them. The antibacterial activities of five compounds against three indicator bacteria were evaluated in this study, and the MIC results showed that compounds 2, 3, and 4 from *A. niger* xj showed definite antibacterial activities against the indicator bacteria except compounds 1 and 5. Among them, compound 4 showed the strongest antibacterial activity against the tested bacteria, especially against RS-2.

Compound 4 (5-hydroxymethyl-2-furancarboxylic acid) is classified as furan ring derivative in the structure of the compound. The research reports that furan ring derivatives have good application prospects in agriculture and medicine, they can be used as an anesthetic in medical treatment, and their anesthetic effect is the same as cocaine anesthetics (Chu, 2013); studies have reported that various furan ring derivatives have antagonistic activity against various pathogens, such as the calcium salt of 2,5-furandicarboxylic acid has antagonistic activity against *Bacillus megaterium* (Lewkowski, 2003), 5-hydroxymethyl has good antagonistic effect on *Alternaria alternata* (Zhang et al., 2019), and 5-(nitrophenyl)-2-furancarboxylic acid aryl ester has different antagonistic activities against *E. coli* and *S. aureus* (Dai et al., 1989). The results of the evaluation of the antimicrobial activity showed that all the tested bacteria were most sensitive to compound 4, and EC-1, T-37, and RS-2 strains were completely inhibited by 31.25, 250, and 15.65 μg/ml, respectively, indicating that it could be applied for biological control. Therefore, it can be judged that furan ring derivatives are a kind of compound with a broad spectrum of activity and potential research value.

Scanning electron microscopy is an effective tool for microstructural analysis, which enables researchers to directly observe the surface morphology of samples. The effect of drugs on the surface structure of bacteria can be directly observed by SEM, and the ultrastructure of cells can be reflected more comprehensively and intuitively. In our study, SEM observation showed that only part of the RS-2 cells treated with compound 4 had the characteristics of apical swelling and irregular cell morphology, and there was no diffusion of cell contents caused by a large area of cell membrane damage. The cell membrane plays an important role in osmotic protective transport and cell biosynthesis (Liu et al., 2018). Some studies suggested that the increases in cell membrane permeability would cause the leakage of various important intracellular components and lead to cell death (Lv et al., 2011). In general, electrical conductivity and leakage of intracellular molecules are used to reflect the effect of a drug on cell membrane permeability. For instance, Lambert et al. (2001) have reported the antibacterial mechanism of oregano essential oil by verifying the membrane permeability and intracellular inorganic ion leakage of *Pseudomonas aeruginosa* and *S. aureus*. The present results showed that compound 4 showed a significant difference in the extracellular conductivity of RS-2 compared to the blank control, which indicated that compound 4 had a certain effect on the membrane permeability of RS-2. The results of relative conductivity and SEM demonstrated that compound 4 had less effect on RS-2 cell membrane and did not cause destructive effects on the structure of cell membrane, and therefore, we speculate that the cell membrane might not be one of the targets of compound 4. Although cell membranes are the targets of many antimicrobial agents, the effects of antimicrobial agents on bacterial cell morphology may be different. Liu et al. (2018) reported that 3-phenyllactic acid treatment severely damaged the cell membrane structure of *Enterobacter cloacae*, resulting in the leakage of intracellular components, which was different from our results. Changes in protein patterns and contents analyzed by SDS-PAGE indicated that the protein synthesis pathway of bacterial cells was affected, and compound 4 might interfere with bacterial cell protein synthesis or induce bacterial protein degradation, which is consistent with the results of Kang et al. (2020). Overall, our results indicated that the RS-2 strain may be disturbed greatly, including cell protein synthesis, as well as intracellular metabolic activity. The antibacterial mechanism of antibacterial compounds against pathogenic bacteria is probably not determined by a specific mechanism, but the result of the combined action of multiple mechanisms (Burt, 2004). Therefore, it is necessary to further study whether compound 4 affects other important intracellular components (DNA, RNA, polysaccharides, etc.) or normal life metabolic pathways, further inhibiting cell growth or causing cell death.

For a long time, there are many strategies to control *R. solanacearum* mainly including chemical pesticides, resistant varieties, and sterile grafting. Rivard et al. (2012) grafted tomatoes with resistant rootstocks to control bacterial wilt (BW) caused by *R. solanacearum*, and the results showed that the incidence of BW in grafted plants decreased by 30–50% compared with that of the non-grafted plants. Manickam et al. (2021) used five BW-resistant eggplant germplasms as rootstocks for grafting tomato, after inoculation with BW, the disease incidence for the grafted plants was 0 to 20%, and the tomato yield of grafted eggplant rootstocks was higher than that of non-grafted tomatoes. Lin et al. (2005) introduced the *Arabidopsis NPR1* gene into a tomato cultivar with resistance to tomato mosaic virus (ToMV), and the results of disease screens against eight important tropical diseases revealed that the transgenic lines exhibited significantly enhanced resistance to bacterial wilt (BW) and fusarium wilt (FW). Chan et al. (2005) introduced the *Arabidopsis thionin* (T*hi2.1*) gene into tomato and the same results were obtained. In addition, studies have found that tobacco can also enhance resistance against bacterial wilt through systemic acquired resistance (Tahir et al., 2017). However, treatment with these agents may cause severe side effects; In contrast, from an environmental point of view, control strategies based on microbial agents and their metabolites against potential pathogens are very attractive and promising. Therefore, obtaining natural bioactive compounds from *A. niger* which are efficient and environmentally friendly is extremely important for controlling bacterial pathogens.

In the early stage of the laboratory, *R. solanacearum* was isolated from susceptible tobacco plants in Dafang county, Guizhou Province, it can infect a few Solanaceae plants, including tobacco, tomato, and potato, and the taxonomic level is phylotype II/sequevar 1 (formerly race 3 biovar 2), which is consistent with the description of Garcia et al. (2019). Tang et al. (2019) developed the microbial agents (*A. niger* effervescent tablets) for tobacco bacterial wilt. Pot experiments proved that the control effect of microbial agents for tobacco bacterial wilt disease reached 92.9% and field experiments proved that the effervescent tablet can effectively promote the growth of tobacco seedlings. The content of effective ingredient of *Aspergillus niger* effervescent tablets is 2 × 10^10^ spores/g, and the cost of applying 30 g effervescent tablets (about 1,000 m^2^) is about 2.69 dollars, which can effectively control bacterial soil-borne diseases (bacterial wilt) and have obvious growth-promoting effects. Fan et al. (2021) took *A. niger* xj spore powder as the effective component of pellet seed coating and investigated the effects of the biocontrol agents on seed germination, seedling growth, and seedling leaf spot disease of sorghum, the results showed that the optimal coating concentration of *A. niger* was 5 × 10^8^ spores/g, which could effectively promote sorghum seed germination and seedling growth, and the control effect on leaf spot disease reached 82.77%.

As previously described, the *A. niger* used in this study is an enzyme production strain approved by the FDA of the United States for food industry. It has the characteristics of vigorous vitality and rapid reproduction, which lays the foundation for the large-scale fermentation of *A. niger* spores. Moreover, the control effect of *A. niger* on *R. solanacearum* is stable and effective, and it is safe and reliable for humans and the environment. The use of antibacterial compounds against *R. solanacearum* will promote the application of *A. niger* in agriculture and further provide a new theoretical basis for the isolation and application of other bioactive metabolites.

## Conclusion

In summary, using conventional natural product separation means, chromatographic column separation technology and combined with activity indicator bacteria for activity tracking, five monomer compounds were successfully isolated from *A. niger* xj, including two new, compounds 3 and 5, and three known compounds: 1, 2, and 4. The results from this study demonstrated that compound 4 displayed notably antimicrobial effects against the indicator bacteria, especially against RS-2. In this study, scanning electron microscopy observation, relative conductivity measurement, and SDS-PAGE analysis showed that the antibacterial mechanism of compound 4 against RS-2 was not causing cell membrane damage of pathogenic bacteria, but a series of reactions caused by the change in intracellular environment, which made the protein could not be synthesized normally, thus affecting the normal life activity of cells and finally leading to cell death. However, a more comprehensive antibacterial mechanism still needs further study. Based on these results, compounds with antimicrobial activity were successfully obtained, which laid a theoretical foundation for the development of biological pesticides and the manufacture of biological agents with very important research value and a wide range of applications.

## Data Availability Statement

The original contributions presented in this study are included in the article/Supplementary Material, further inquiries can be directed to the corresponding author/s.

## Author Contributions

LW and QZ participated in the design and experiments, data acquisition and analysis, and drafted and revised the manuscript. KG designed the experiments and contributed to data acquisition and helped to draft the manuscript. YuX, AX, and TH contributed to data analysis, provided software, and helped to revise the manuscript. ZL conceived the idea and participated in the design and contributed to data analysis and interpretation and helped to revise the manuscript critically. SM and YaX contributed to data analysis and the determination of compounds and helped to revise the manuscript. All authors read and approved the final manuscript.

## Conflict of Interest

The authors declare that the research was conducted in the absence of any commercial or financial relationships that could be construed as a potential conflict of interest.

## Publisher’s Note

All claims expressed in this article are solely those of the authors and do not necessarily represent those of their affiliated organizations, or those of the publisher, the editors and the reviewers. Any product that may be evaluated in this article, or claim that may be made by its manufacturer, is not guaranteed or endorsed by the publisher.
