# Supplementary material for: Isolation of Bioactive Compounds, Antibacterial Activity, and Action Mechanism of Spore Powder From Aspergillus niger xj
Source: Front Microbiol. 2022 Jul 11;13:934857. doi: 10.3389/fmicb.2022.934857 (PMC9309528; doi:10.3389/fmicb.2022.934857)
Supplement: Supplementary file 1 [file Data_Sheet_1.docx]

***Supplementary Materials***


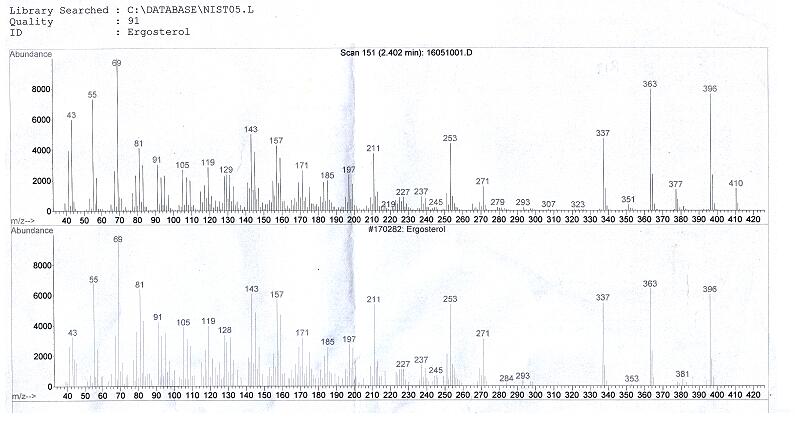


**Supplementary Figure 1**. The EI-MS spectrum of ergosterol (compound **1**).

**
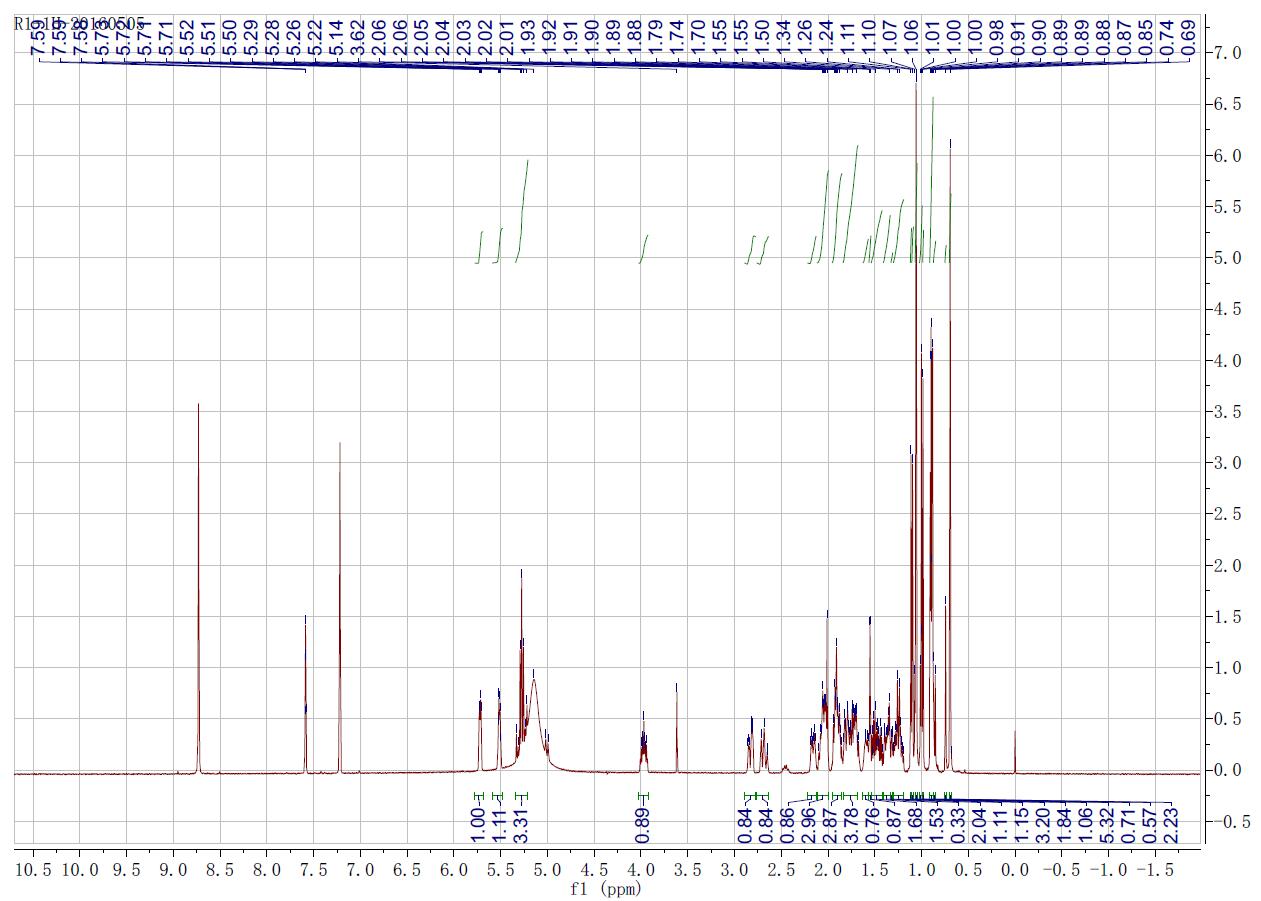
**

**Supplementary Figure 2**. The ^1^H-NMR spectrum of ergosterol (compound **1**).


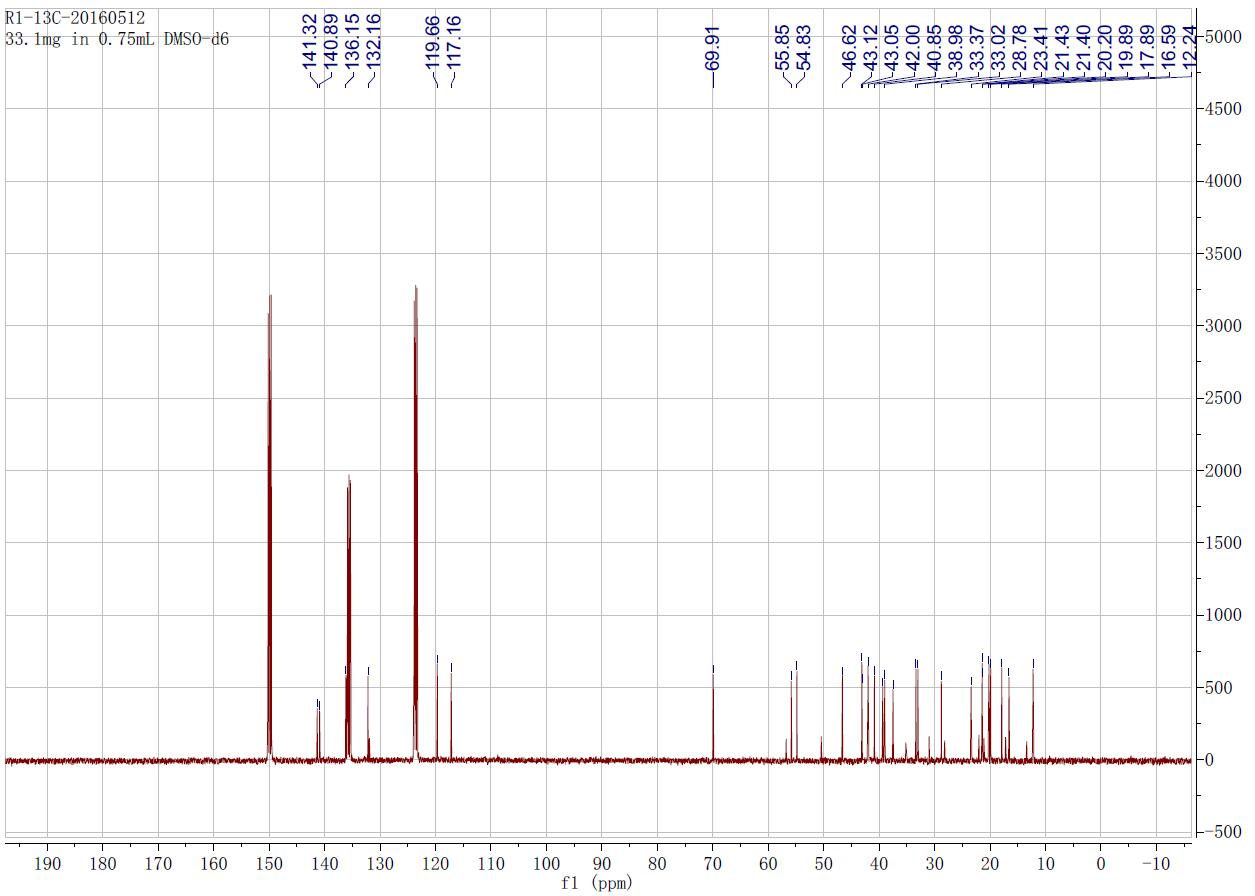


**Supplementary Figure 3**. The ^13^C-NMR spectrum of ergosterol (compound **1**).


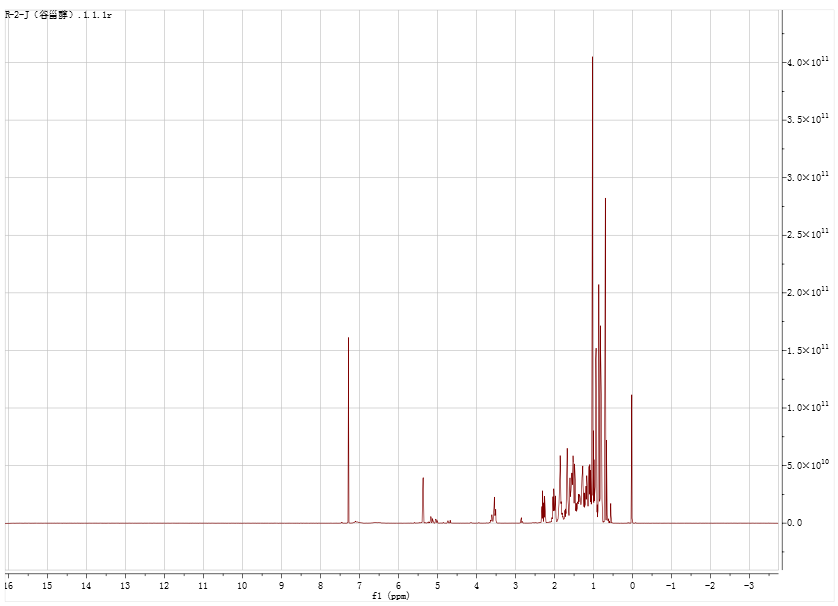


**Supplementary Figure 4**. The ^1^H-NMR spectrum of β-sitosterol (compound **2**).


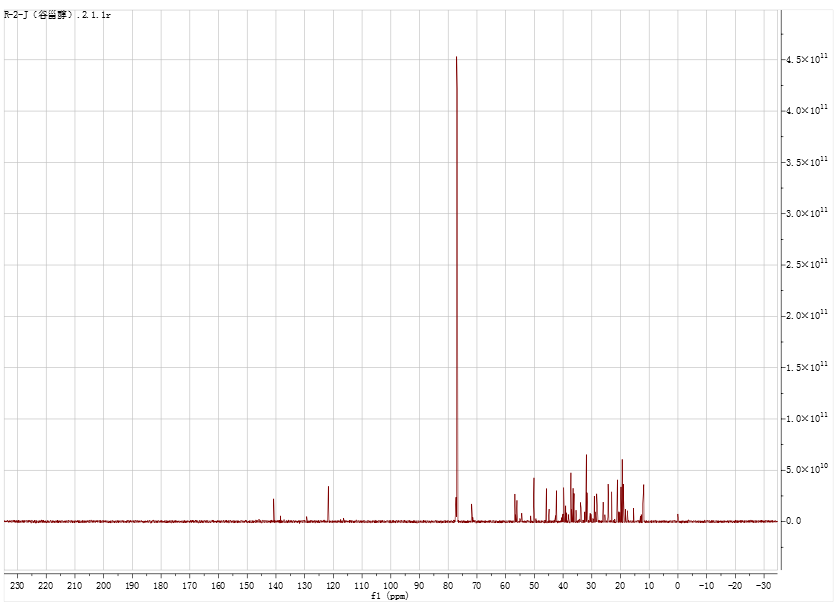


**Supplementary Figure 5**. The ^13^C-NMR spectrum of β-sitosterol (compound **2**).


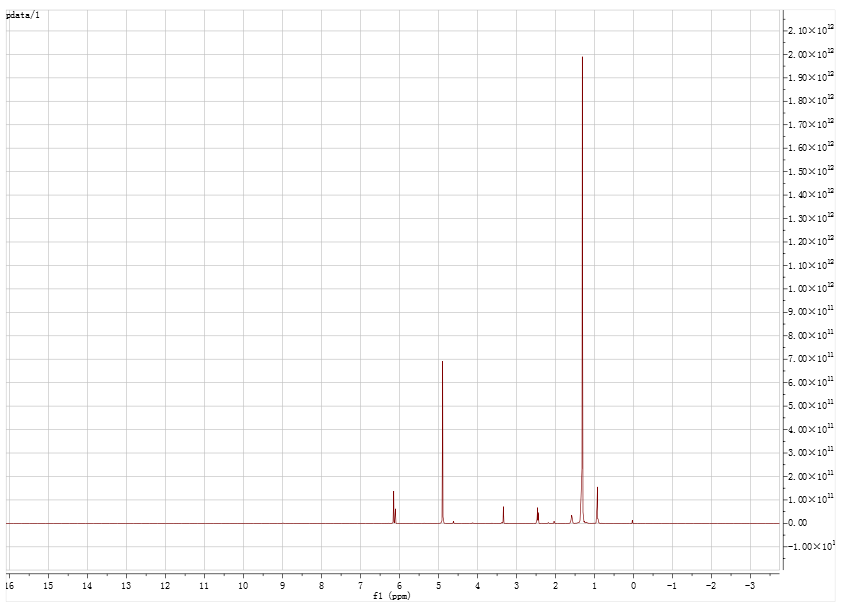


**Supplementary Figure 6**. The ^1^H-NMR spectrum of 5-pentadecylresorcinol (compound **3**).


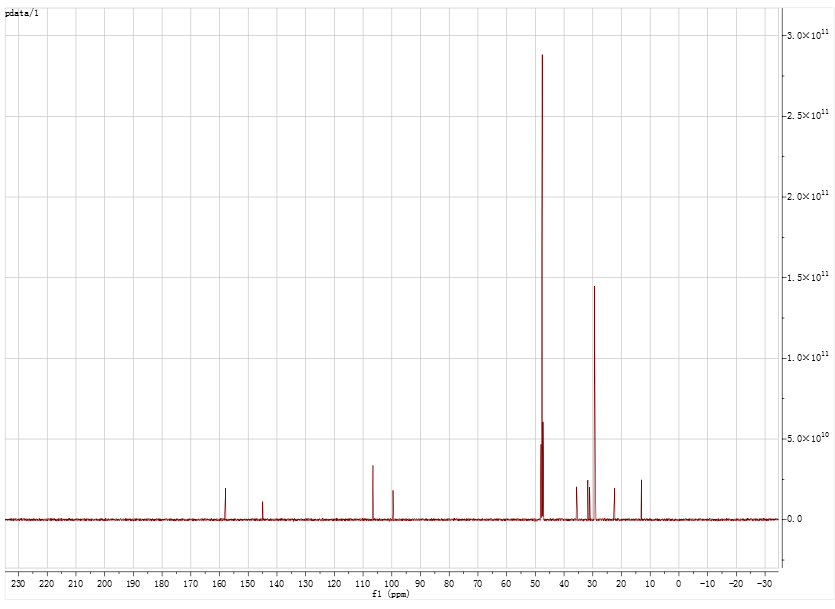


**Supplementary Figure 7**. The ^13^C-NMR spectrum of 5-pentadecylresorcinol (compound **3**).


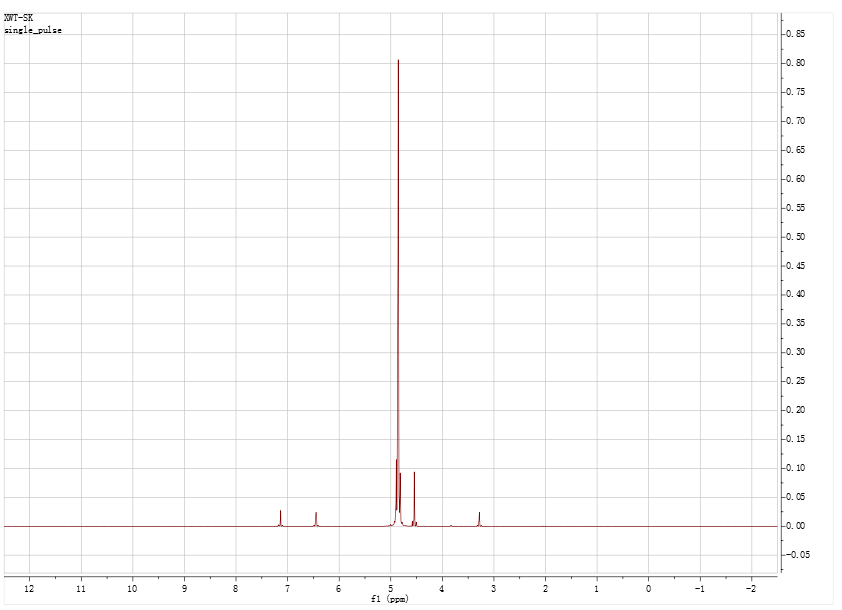


**Supplementary Figure 8**. The ^1^H-NMR spectrum of 5-hydroxymethyl-2-furancarboxylic acid (compound **4**).


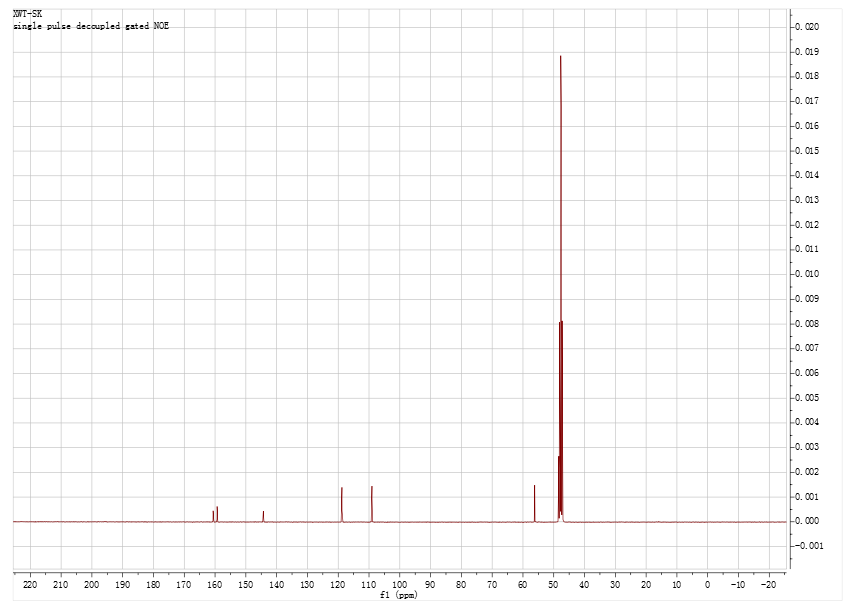


**Supplementary Figure 9**. The ^13^C-NMR spectrum of 5-hydroxymethyl-2-furancarboxylic acid (compound **4**).


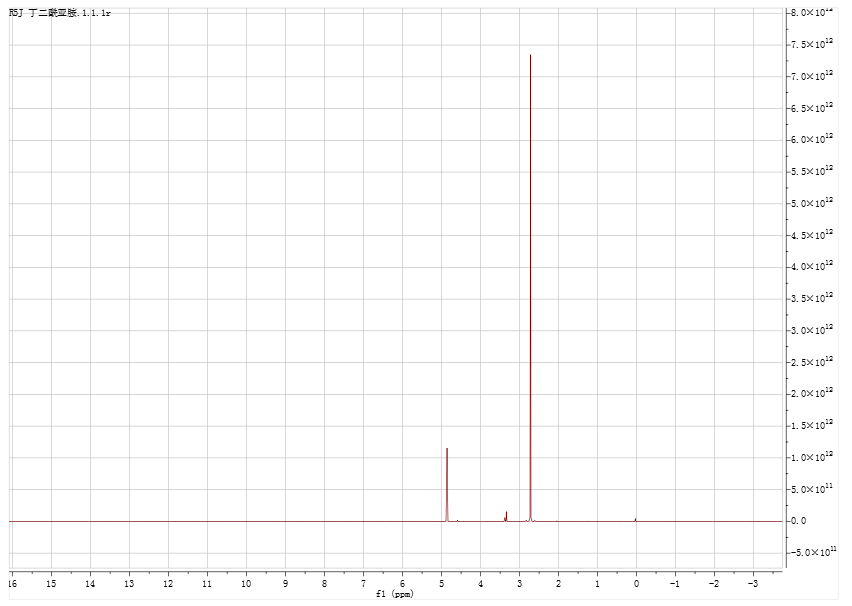


**Supplementary Figure 10**. The ^1^H-NMR spectrum of succinimide (compound **5**).


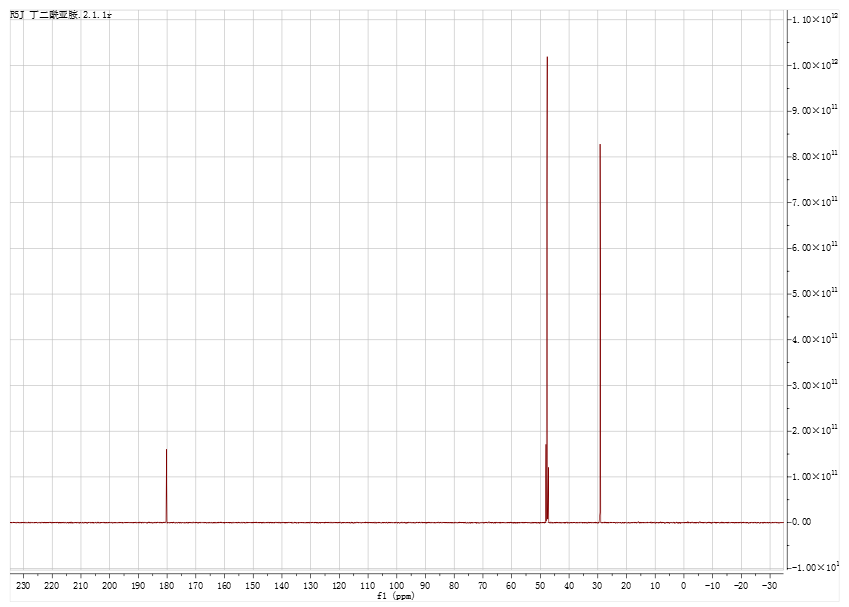


**Supplementary Figure 11**. The ^13^C-NMR spectrum of succinimide (compound **5**).
